# Diffusion of an innovation: growth in video capsule endoscopy in the U.S. Medicare population from 2003 to 2019

**DOI:** 10.1186/s12913-022-07780-2

**Published:** 2022-03-31

**Authors:** Andrew J. Read, Michael D. Rice, Jason R. Baker, Akbar K. Waljee, Sameer D. Saini

**Affiliations:** 1 Division of Gastroenterology and Hepatology, University of Michigan, Ann Arbor, MI USA; 2 Institute for Healthcare Policy and Innovation, University of Michigan, Ann Arbor, MI USA; 3 Atrium Health, Carolinas Medical Center, Charlotte, NC USA; 4 VA HSR&D Center for Clinical Management Research, Ann Arbor, MI USA

**Keywords:** Video capsule endoscopy, Small intestine, Small bowel, Diffusion of innovation, Endoscopy, Endoscopic technology

## Abstract

**Background:**

Video capsule endoscopy (VCE), approved by the U.S. Food and Drug Administration (FDA) in 2001, represented a disruptive technology that transformed evaluation of the small intestine. Adoption of this technology over time and current use within the U.S. clinical population has not been well described.

**Methods:**

To assess the growth of capsule endoscopy within the U.S. Medicare provider population (absolute growth and on a population-adjusted basis), characterize the providers performing VCE, and describe potential regional differences in use. Medicare summary data from 2003 to 2019 were used to retrospectively analyze capsule endoscopy use in a multiple cross-sectional design. In addition, detailed provider summary files were used from 2012 to 2018 to characterize provider demographics.

**Results:**

VCE use grew rapidly from 2003 to 2008 followed by a plateau from 2008 to 2019. There was significant variation in use of VCE between states, with up to 10-fold variation between states (14.6 to 156.1 per 100,000 enrollees in 2018). During this time, the adjusted VCE use on a population-adjusted basis declined, reflecting saturation of growth.

**Conclusions:**

Growth of VCE use over time follows an S-shaped diffusion of innovation curve demonstrating a successful diffusion of innovation within gastroenterology. The lack of additional growth since 2008 suggests that current levels of use are well matched to overall population need within the constraints of reimbursement. Future studies should examine whether this lack of growth has implications for access and healthcare inequities.

**Supplementary Information:**

The online version contains supplementary material available at 10.1186/s12913-022-07780-2.

## Background

Video capsule endoscopy (VCE) was first approved by the U.S. Food and Drug Administration (FDA) in 2001 and has since become an integral tool in evaluation of the small intestine [1–3]. Prior to VCE, endoscopic visualization of the deeper small intestine was time-consuming, invasive, and limited to specialty referral centers [4]. VCE has been described as a “disruptive technology,” as it dramatically transformed management of gastrointestinal (GI) bleeding and other small bowel diseases, including Crohn’s disease, celiac disease and hereditary polyposis syndromes [1, 5, 6]. Diagnostic visualization of the entirety of the small bowel could now be achieved by swallowing a pill, rendering other techniques such as Sonde enteroscopy (a diagnostic procedure relying on passive advancement of an enteroscope by peristalsis) obsolete in comparison [1, 4].

But how quickly does a technologic advancement such as the introduction of VCE spread among practicing gastroenterologists? The theory of Diffusion of Innovations, developed by the sociologist Everett Rogers, has been applied to models of technologic diffusion in medicine, and may serve as a model to understand the growth and diffusion of VCE technology [7–11]. Despite the availability of VCE technology and established guidelines for use, we have limited data on how frequently VCE is performed, regional performance of VCE, growth trends in VCE use over time, and providers who perform VCE [12–14]. Prior research has attempted to quantify the use of other endoscopic procedures such as esophagogastroduodenoscopy (EGD) and colonoscopy, but these studies have not examined VCE [15–17]. VCE has become an important component of U.S. gastroenterology fellowship training programs, but the use and availability of VCE in routine clinical practice in the U.S. is not well described [18–20].

Our objective was to understand the adoption of VCE by U.S. Medicare providers over time, describe the characteristics of providers performing VCE, and determine the regional availability of VCE within the U.S. To do this, we conducted a retrospective cross-sectional evaluation of administrative data from Medicare Part B from 2003 to 2019.

## Methods

We hypothesized that after its introduction, VCE usage among the Medicare-eligible population would rapidly grow, but then gradually decelerate, after a sufficient number of providers added VCE to their clinical practice, saturating the demand for approved VCE indications. Our primary outcome was the number of VCEs performed per year in Medicare patients, and the secondary outcome was the number of VCEs performed on a population-adjusted basis, controlled for changes in Medicare enrollment over time.

Data from publicly available Medicare Part B datasets and Medicare enrollment were obtained from the Centers for Medicare & Medicaid Services (CMS) website (CMS.gov) [21]. The U.S. Medicare program includes over 60 million adults, primarily those aged 65 and over (84%), but also certain individuals under age 65 with specific disabilities and conditions, including end stage renal disease on dialysis [21, 22]. Three separate Medicare Part B datasets were used: (1) annual national summary data files, containing total counts of procedures by Current Procedural Terminology (CPT) codes from 2003 to 2019; (2) annual summary data by states from 2012 to 2018; (3) detailed individual provider-level billing data from 2012 to 2018. The individual provider level file includes data on providers who performed more than 10 of an individual CPT procedure code in an individual year. In addition, we used the publicly accessible National Plan and Provider Enumeration System (NPPES) file from CMS.gov, which contains data on a provider’s gender, provider’s specialty taxonomy code(s), and date of National Provider Identification (NPI) enumeration. We linked this to the Medicare Part B file by NPI numbers to improve characterization of the providers’ demographics. We also obtained the annual number of Medicare enrollees nationally and by state from CMS.gov. We also used the Centers for Disease Control and Prevention (CDC) National Center for Health Statistics 2013 (NCHS) Urban-Rural Classification Scheme for Counties to classify each provider’s practice environment by degree of urbanization based on each provider’s address [23].

Using the national annual Medicare Part B summary data, we determined the number of VCE procedures performed per year and calculated a population-adjusted rate of use. The absolute number of VCEs performed per year were tabulated using the temporary CPT code G0262 for 2003 to 2004 and permanent CPT code 91110 for 2004–2019 [24]. To control for changes in size of the Medicare enrolled population during the study period, we calculated a ratio of the number of VCEs performed per 100,000 Medicare enrollees per year nationally for 2003–2019. We used the annual state summary files from 2012 to 2018 to similarly calculate a ratio of VCEs/100,000 Medicare enrollees for each state.

We calculated descriptive statistics on the providers who performed > 10 VCE studies per year using data from the detailed provider-level information files (2012–2018) linked to the NPPES and NCHS files. These files included each provider’s gender, date of NPI number enumeration, specialty information by taxonomy codes, and mailing address. As provider age or length of clinical practice is not explicitly available within these datasets, we determined an approximate duration of clinical practice by calculating the difference between the year of evaluation (e.g., 2018) and the year of NPI number enumeration. We determined each provider’s medical specialty using the provider type listed within the Medicare Part B file and, in cases of ambiguity, used the detailed healthcare provider taxonomy codes from the NPPES file (linked by NPI numbers) to further improve specificity. For example, in cases of providers listed as “Internal Medicine” providers, we further defined them as gastroenterologists if their taxonomy codes were “207RG0100X” (gastroenterology), “207RI0008X” (hepatology), or “207RT0003X” (transplant hepatology). In addition, we categorized each provider’s practice setting by population density (e.g., large central metropolitan) using data from the National Center for Health Statistics 2013 (NCHS) classification system based on each provider’s address.

We characterized providers’ practice type by examining other procedure codes performed by those providers. For example, we categorized providers as advanced endoscopists if they performed more than 10 endoscopic retrograde cholangiopancreatography (ERCP) or endoscopic ultrasound (EUS) procedures within the year, using corresponding CPT codes. Using a similar method, we identified providers who performed general endoscopy (esophagogastroduodenoscopy or colonoscopy) or who performed GI motility studies using CPT codes.

This study was considered exempt by the University of Michigan Institutional Review Board review as all data are publicly available and without patient-level identifiers. Statistical analysis was performed using SAS 9.4 (SAS Institute, Cary, NC) and R Core Team (2020). Graphs were produced using GraphPad Prism 8.0 (GraphPad Software, San Diego, CA) and maps using R Core Team (2020).

## Results

### Growth of VCE

**Fig. 1:**
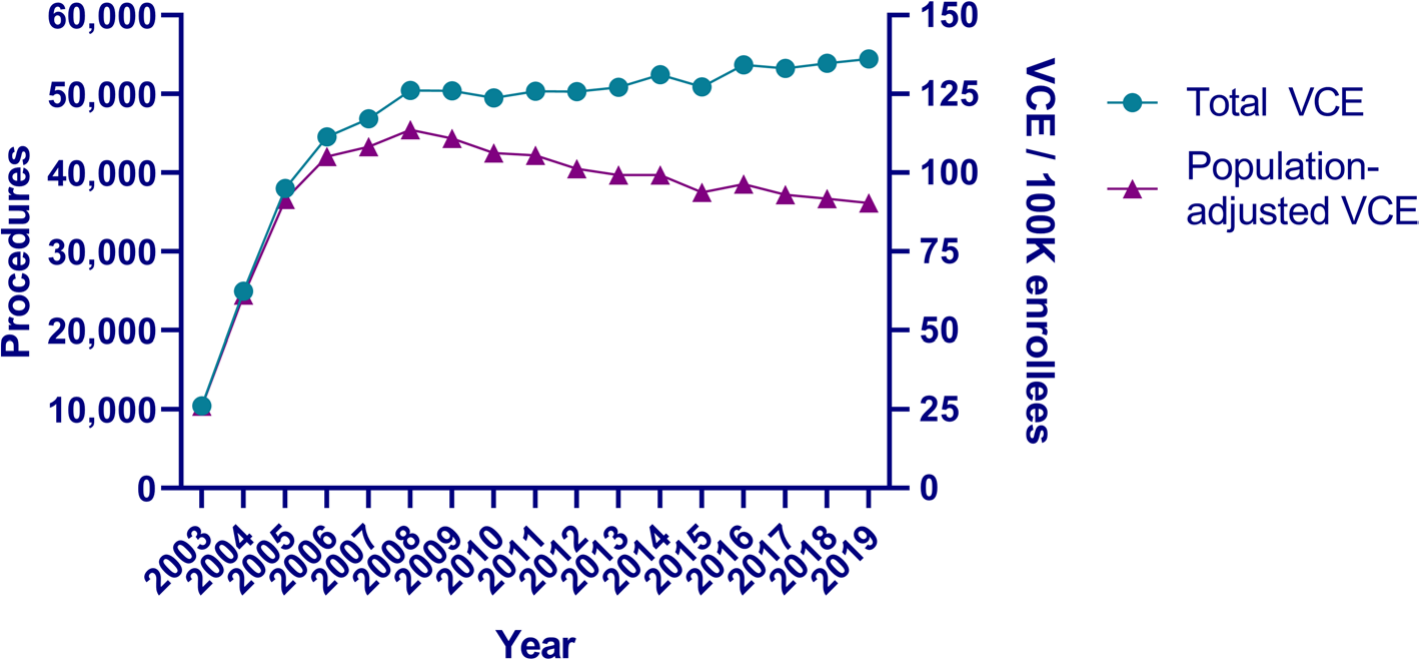
Number of video capsule endoscopies (VCEs) performed in Medicare population 2003–2019. Total number of VCEs performed (left y-axis) and adjusted number of VCEs / 100 K Medicare enrollees per year (right y-axis). Total VCE performed was calculated by Temporary CPT Code G0262 in 2003; sum of Temporary CPT Code G0262 + dedicated CPT Code 91110 in 2004; and CPT Code 91110 for 2005–2015

The use of VCE in Medicare Part B increased rapidly after introduction: increasing to 10,483 in 2003 (first year covered in Medicare with a temporary CPT code), more than doubling in 2004 to 24,950 (first year of a dedicated CPT code) and increasing to 38,031 in 2005 (Fig. 1). While the number of procedures performed per year continued to increase, the rate of increase decelerated and by 2008 there were 50,478 procedures performed. Over the subsequent 11 years, the absolute number of VCE procedures performed per year in the Medicare population remained relatively stable with some annual fluctuations (range: 49,522 - 54,467). However, the relative number of VCEs performed per 100,000 Medicare enrollees declined over this period, from a peak of 113.7 per 100,000 enrollees in 2008 to 90.4 per 100,000 enrollees in 2019 (Fig. 1).

### VCE provider characteristics

We characterized the demographics and practice locations of providers performing more than 10 VCEs/year in 2018 by gender, practice setting (size of community by NCHS classification), approximate years in clinical practice, and specialty type (Table 1). The majority of VCE providers were male (89.4%, *n* = 1142/1278) and 97.6% (*n* = 1247/1278) were gastroenterologists. The majority had been in practice for 10 or more years (90.8%, *n* = 1160/1278) and nearly all also performed general endoscopic procedures (EGD or colonoscopy) (98.5%, *n* = 1259/1278). A minority of the providers (20.3%, *n* = 260/1278) performed advanced endoscopic procedures (EUS or ERCP), and 18.9% (*n* = 242/1278) performed GI physiology studies. The majority of these providers were in higher density population regions, with 53.1% (*n* = 678/1278) in large central metro or large fringe metro areas.

**Table 1:** Characteristics of providers performing > 10 video capsule endoscopy (VCE) procedures/year in Medicare Part B in 2018. Gender based on National Plan and Provider Enumeration System (NPPES) file. Practice setting determined by provider address and National Center for Health Statistics 2013 (NCHS) Urban-Rural Classification Scheme for Counties

Characteristic	***n*** = 1278
**Provider Specialty**	**no. (%)**
Gastroenterology	1247 (97.6%)
Surgery	13 (1.0%)
Internal / Family Medicine	12 (0.9%)
Other	6 (0.5%)
**Practice Duration**	**no. (%)**
< 5 years	6 (0.5%)
5–10 years	112 (8.8%)
10 or more years	1160 (90.8%)
**Gender**	**no. (%)**
Male	1142 (89.4%)
Female	136 (10.6%)
**Clinical Practice Pattern**	**no. (%)**
General Endo (EGD or Colonoscopy)	1259 (98.5%)
Advanced Endo (EUS or ERCP)	260 (20.3%)
GI Physiology Studies (Esophagus/Motility)	242 (18.9%)
**VCE Procedures by Provider**	
Median	16
IQR	13–24
Maximum	235
**Other Procedures by Provider**	
Median	495.5
IQR	324–745
Maximum	3164
**Practice Setting (NCHS Classification)**	**no. (%)**
Large central metro (Pop. 1,000,000+)	348 (27.2%)
Large fringe metro	330 (25.8%)
Medium metro (Pop. 250,000–999,999)	315 (24.7%)
Small metro	182 (14.2%)
Micropolitan (10,000–49,999)	95 (7.4%)
Non-core (Rural)	8 (0.6%)

### Regional availability of VCE

**Fig. 2:**
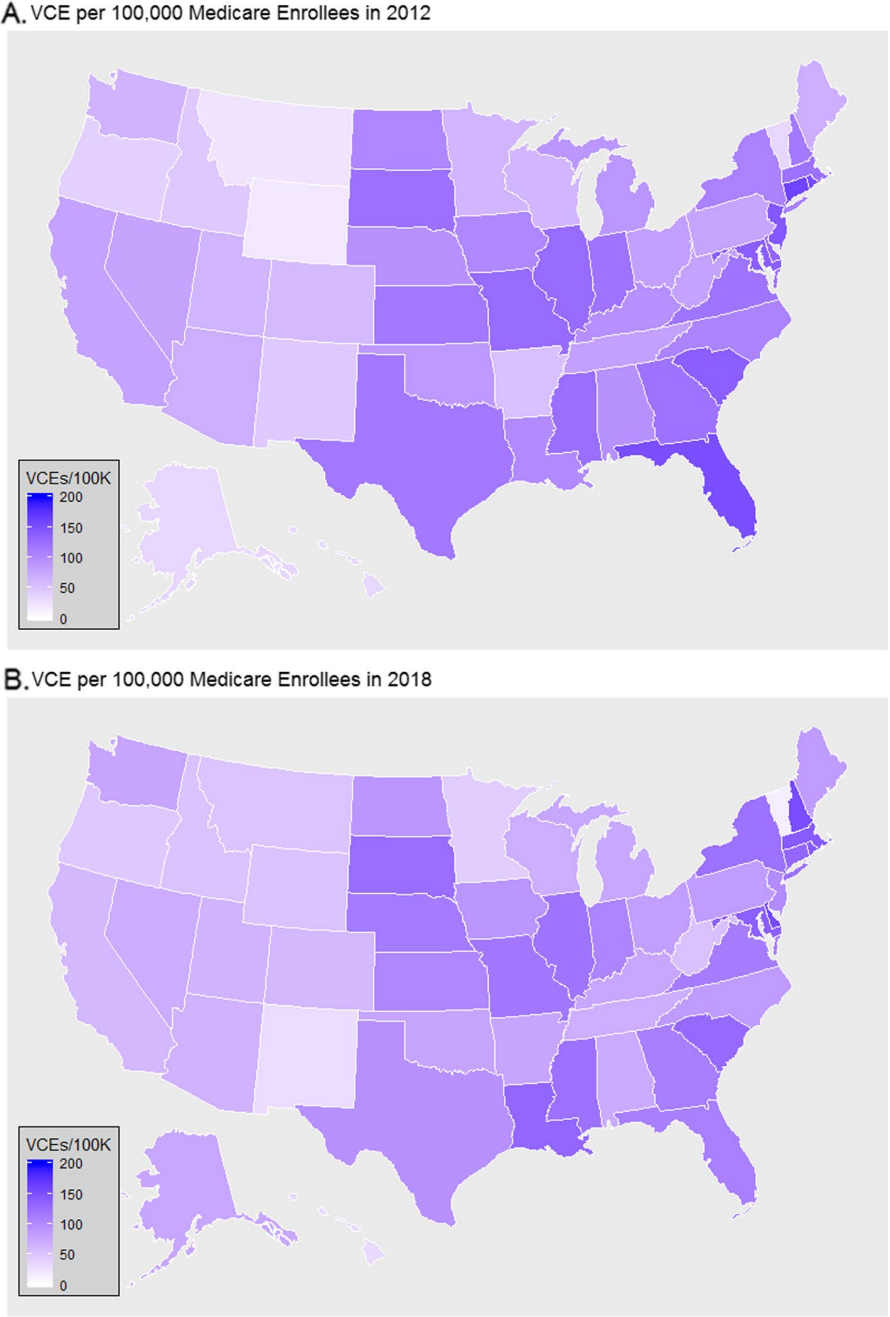
Video Capsule Endoscopy (VCE) use by state over time (**A**. 2012, **B**. 2018). Rates are calculated per 100,000 Medicare Part B enrollees by state per year. Darker colors indicate more VCE procedures performed per enrolled population. (Maps generated in R with ggplot2 library.) Data for 2012–2018 available in Supplemental Table 1

Using the annual Medicare B CPT summary data by state (2012–2018), we calculated the ratio of VCEs performed per 100,000 Medicare enrollees by state (Fig. 2). We found up to 10-fold variation in VCE utilization between states: 14.6 per 100,000 enrollees (Vermont) to 156.1 per 100,000 enrollees (Delaware) in 2018 (Supplemental Table 1). Regionally, this demonstrated a relatively lower use of VCE on a per enrollee basis in western states within the U.S.

## Discussion

This study demonstrates a rapid uptake of VCE use in Medicare patients upon introduction, followed by a gradual slowing and plateau of use in more recent years (Fig. 1). When adjusted for the increasing number of Medicare enrollees over time, there has been a slight decline in more recent years on a population-adjusted basis. This rapid uptake can be best understood by the theory of diffusion of innovation, first described in sociology [7], but has been applied to multiple innovations in medicine, including telehealth and cardiac stenting [8, 10, 11, 25–28]. How could there have been such rapid adoption and diffusion of this technology? As a “disruptive technology,” VCE had substantial advantages over existing technologies, allowing evaluation of the entire small bowel intestine non-operatively, without radiation, with improved comfort for the patient (requiring only swallowing a large capsule), and improved scheduling for physicians (allowing them to interpret the study asynchronously).

As with other diffusion of innovation curves, we anticipated the growth rate would decelerate over time, once the late majority adopted the technology, but we did not expect to discover that the population-adjusted use of VCE would decline (Fig. 1). There are several potential reasons for this observation. First, as there are finite indications for VCE, the plateau observed in Fig. 1 likely represents a relatively stable number of providers/practices offering VCE, and as a result, there may be limited growth potential within an existing region. Second, while VCE was a “disruptive technology” when introduced, subsequent advances within capsule endoscopy have been more “sustaining innovations” rather than “disruptive” (e.g., incremental improvements such as improved resolution, increased field of view) [29]. Other capsule endoscopy procedures, such as esophageal capsule endoscopy, are based on the same fundamentals of small bowel VCE, and have had considerably less adoption due to relative ease of evaluation of those regions of the GI tract using esophagogastroduodenoscopy (EGD) (Supplemental Table 2). Finally, competing diffusion of innovations of other technologies may also have reduced the demand for VCE for some indications: e.g., MRI or CT enterography (no risk of capsule retention, an increased concern for patients with prior small bowel surgeries or stenotic Crohn’s disease) and device-assisted enteroscopy (single or double balloon enteroscopy, with the potential for diagnostic biopsies and possible therapeutic interventions deep within the small intestine). Future innovations in capsule endoscopy including colon capsule endoscopy (with a dedicated CPT code 91113 created in 2021, after this study period) [13, 30] and magnetically controlled capsule endoscopy [31] may further reshape the relative use of diagnostic capsule studies vs. potentially therapeutic endoscopic studies.

In addition, within the U.S. healthcare system, there are financial considerations for individual physicians and clinical practices in adopting a new technology. When first introduced, VCE quickly dominated existing technologies, creating pressure on clinicians and practices to offer this technology or potentially lose business to competitors. For clinical practices, there was an initial upfront adoption cost to purchase the reusable radiofrequency receiver devices, with additional (lower) per-item costs for the disposable capsules. Thus, once purchased, there is a financial incentive to perform more procedures to recoup the investment cost and then generate additional profit. For clinicians within a fee-for-service system, as exists in much of the U.S., the professional fees associated with a procedure may affect their decision to adopt a new procedure. With the asynchronous nature of VCE procedures (which are performed and interpreted separately), for some clinicians, this could represent new revenue if these procedures were interpreted outside of existing clinical schedules. From a financial standpoint, during the course of the study, there was decrease in valuation in physician relative value units (RVUs) from 3.64 RVUs in 2016 (comparable to the current 3.56 RVUs for a colonoscopy with biopsy, CPT 45380) to 2.49 in 2017 (the same value as a diagnostic push enteroscopy, CPT 44360). The time for interpretation of VCE studies likely varies considerably based on study and provider characteristics, and based on their own preferences, some clinicians may elect to perform other procedures for greater reimbursement.

Other technologic innovations in healthcare have been examined using the diffusion of innovations framework and provide additional insights into why some innovations achieve successful diffusion while others are abandoned. While there may be a publication bias toward successful diffusion of innovations, telemedicine/telehealth provides a unique example about an initial failure and later successful diffusion. When first introduced in the 1960s, the technology failed to achieve widespread diffusion, in part because computing and communication technology had not yet achieved the levels of connectivity that would be needed for success [32]. With improvements in information technology, these technologies were later gradually adopted in the 1990s for teleradiology (allowing remote review of radiologic studies) [25] and in the 2000s with ICU telemedicine (allowing for remote critical care coverage) [26]. These applications allowed specialty physicians to provide additional clinical services from afar, providing needed services to smaller hospitals without in-house specialty access. Direct physician to patient telehealth services also gradually increased, [33] but were massively accelerated by the disruptive nature of the COVID-19 pandemic, with some practices shifting entirely to telemedicine [34, 35]. However, pre-existing structural barriers in broadband internet access that predated the COVID-19 pandemic [36–38] limited the adoption of video telemedicine among all groups equally [39–41]. The same risks may apply with diffusion of technologic innovations within gastroenterology, where newer, highly specialized procedures may not be offered in all regions, leading patients either not to have access to the technologies locally, requiring either forgoing the treatment or long travel to subspecialty centers. In this context, technologies such as VCE that can allow for asynchronous physician interpretation of diagnostic studies, were well suited for a transition toward virtual care. However, the pre-existing variation in VCE usage per Medicare enrollee we identified across states suggests that there is either endoscopic underuse/overuse of this technique or significant disparities in access to this technology.

There are some limitations in the use of this dataset. First, this dataset contains information only on providers, not patients, and therefore more detailed inferences about the specific indications on patient characteristics of those undergoing these procedures is unknown from these data. Second, the detailed provider information was limited to only those providers who performed more than 10 VCEs per year. Thus, the absence of a provider within these data does necessarily mean they do not perform VCEs: they may simply perform fewer VCEs or have limited Medicare patients. To further enrich these data sources, we attempted to gain additional insights about the practice environments using other publicly available datasets, including NPI enumeration dates and NCHS Classifications. One limitation of NPI numbers for this purpose is that they were not required until 2007, thus leading to a large influx of NPI numbers at that time. In addition, to minimize potential bias by censoring of providers who performed 10 or fewer procedures from the individual provider dataset, we also used the state and national datasets which included all billed procedures within a given year. Additional limitations of the methods include the lack of clinical outcomes data (uncertain impact of the new technology on patient care), and the possibility that the diffusion of innovation curve noted within this Medicare population may differ from the broader U.S. population as whole. Future work could evaluate the clinical outcomes and uptake in non-Medicare populations.

## Conclusions

This paper demonstrates that VCE spread rapidly within the U.S. by patterns previously described, fitting the model of a diffusion of innovation. Limitations of this study include the absence of clinical indications and associated outcome variables. Nonetheless, this paper provides important insights into the rate of diffusion of a transformational technology and demonstrates that gastroenterologists (and patients) are open to new technologies that have the potential to rapidly transform clinical practice.

## Supplementary Information

**Additional file 1: Table S1.** Small bowel Video Capsule Endoscopy (VCE) use by state over time (2012–2018), calculated per 100,000 Medicare Part B enrollees by state per year (2012–2018).

**Additional file 2: Table S2.** Performance of esophageal capsule endoscopy (CPT 91111) within the Medicare population 2007–2019.

## Data Availability

The datasets analyzed during the current study are available in the Centers for Medicare and Medicaid Services Research, Statistics, Data & Systems repository, https://www.cms.gov/Research-Statistics-Data-and-Systems/Research-Statistics-Data-and-System.
