# Supplementary material for: Diffusion of an innovation: growth in video capsule endoscopy in the U.S. Medicare population from 2003 to 2019
Source: BMC Health Serv Res. 2022 Mar 31;22:425. doi: 10.1186/s12913-022-07780-2 (PMC8969398; doi:10.1186/s12913-022-07780-2)
Supplement: Supplementary file 1 — Additional file 1: Table S1. Small bowel Video Capsule Endoscopy (VCE) use by state over time (2012–2018), calculated per 100,000 Medicare Part B enrollees by state per year (2012–2018). [file 12913_2022_7780_MOESM1_ESM.docx]

**Supplemental Table 1.** Small bowel Video Capsule Endoscopy (VCE) use by state over time (2012-2018), calculated per 100,000 Medicare Part B enrollees by state per year (2012-2018).

| State | 2012 | 2013 | 2014 | 2015 | 2016 | 2017 | 2018 |
| --- | --- | --- | --- | --- | --- | --- | --- |
| ALABAMA | 95.1 | 95.6 | 99.8 | 91.0 | 90.4 | 80.7 | 72.2 |
| ALASKA | 34.3 | 59.0 | 81.4 | 68.1 | 99.0 | 71.8 | 75.9 |
| ARIZONA | 69.9 | 68.9 | 69.1 | 73.2 | 69.1 | 69.6 | 66.1 |
| ARKANSAS | 53.9 | 53.1 | 55.0 | 59.3 | 64.8 | 54.0 | 76.2 |
| CALIFORNIA | 78.8 | 74.9 | 74.9 | 64.1 | 63.6 | 63.7 | 60.9 |
| COLORADO | 59.1 | 62.4 | 53.4 | 57.4 | 63.0 | 62.9 | 60.6 |
| CONNECTICUT | 161.3 | 151.7 | 152.7 | 145.4 | 156.4 | 138.2 | 131.5 |
| DELAWARE | 130.7 | 166.4 | 158.3 | 141.9 | 148.8 | 165.3 | 156.1 |
| DISTRICT OF COLUMBIA | 105.5 | 106.0 | 76.7 | 69.4 | 101.6 | 117.5 | 107.0 |
| FLORIDA | 152.4 | 147.0 | 138.2 | 126.3 | 120.9 | 117.6 | 110.0 |
| GEORGIA | 121.0 | 110.6 | 117.7 | 112.1 | 119.6 | 118.6 | 110.8 |
| HAWAII | 33.4 | 28.7 | 29.1 | 29.4 | 37.3 | 43.2 | 31.8 |
| IDAHO | 46.5 | 51.9 | 55.4 | 47.2 | 48.3 | 60.1 | 51.5 |
| ILLINOIS | 127.3 | 124.5 | 125.1 | 114.7 | 111.9 | 113.1 | 119.7 |
| INDIANA | 120.2 | 114.8 | 115.0 | 109.3 | 107.5 | 108.9 | 105.6 |
| IOWA | 102.7 | 99.6 | 83.1 | 86.1 | 89.8 | 95.6 | 90.5 |
| KANSAS | 116.1 | 126.2 | 118.6 | 102.4 | 114.1 | 107.3 | 103.6 |
| KENTUCKY | 95.6 | 99.7 | 102.0 | 93.1 | 86.9 | 81.1 | 75.6 |
| LOUISIANA | 100.0 | 104.0 | 117.6 | 123.1 | 129.6 | 114.3 | 131.7 |
| MAINE | 69.5 | 70.8 | 73.4 | 95.9 | 108.2 | 95.0 | 85.1 |
| MARYLAND | 138.2 | 142.7 | 137.3 | 123.4 | 133.8 | 137.5 | 137.8 |
| MASSACHUSETTS | 121.9 | 126.7 | 133.3 | 137.9 | 150.4 | 141.6 | 141.6 |
| MICHIGAN | 89.4 | 91.1 | 82.4 | 84.5 | 80.7 | 79.5 | 75.0 |
| MINNESOTA | 63.3 | 52.6 | 51.5 | 46.5 | 47.8 | 41.9 | 41.8 |
| MISSISSIPPI | 124.7 | 120.5 | 126.2 | 116.9 | 109.8 | 117.6 | 122.2 |
| MISSOURI | 127.5 | 111.6 | 120.7 | 115.9 | 119.7 | 115.3 | 116.5 |
| MONTANA | 22.6 | 29.2 | 37.0 | 35.8 | 28.8 | 57.1 | 50.7 |
| NEBRASKA | 94.7 | 95.5 | 91.3 | 94.3 | 103.7 | 95.9 | 113.3 |
| NEVADA | 78.4 | 75.7 | 75.0 | 73.0 | 71.2 | 60.9 | 70.5 |
| NEW HAMPSHIRE | 115.2 | 129.4 | 127.4 | 141.5 | 175.5 | 168.0 | 154.0 |
| NEW JERSEY | 144.3 | 143.7 | 130.4 | 121.2 | 124.4 | 102.3 | 98.9 |
| NEW MEXICO | 45.5 | 41.9 | 46.6 | 36.3 | 26.5 | 22.2 | 30.0 |
| NEW YORK | 108.1 | 106.6 | 109.7 | 110.3 | 116.3 | 119.0 | 122.4 |
| NORTH CAROLINA | 106.2 | 105.3 | 97.5 | 94.7 | 96.2 | 92.9 | 84.9 |
| NORTH DAKOTA | 103.3 | 88.0 | 88.9 | 84.7 | 78.6 | 106.4 | 90.1 |
| OHIO | 81.7 | 79.6 | 83.3 | 72.1 | 80.4 | 82.8 | 82.9 |
| OKLAHOMA | 88.0 | 84.1 | 85.9 | 80.1 | 77.4 | 68.0 | 77.5 |
| OREGON | 39.6 | 43.8 | 46.9 | 37.5 | 39.6 | 43.6 | 46.0 |
| PENNSYLVANIA | 77.4 | 79.7 | 80.9 | 83.0 | 82.2 | 83.4 | 84.8 |
| RHODE ISLAND | 155.7 | 132.4 | 144.8 | 144.2 | 160.1 | 148.3 | 142.4 |
| SOUTH CAROLINA | 138.5 | 136.0 | 134.2 | 136.2 | 131.8 | 117.7 | 128.6 |
| SOUTH DAKOTA | 123.1 | 160.8 | 152.2 | 130.0 | 120.1 | 127.0 | 125.8 |
| TENNESSEE | 81.3 | 77.3 | 77.1 | 73.3 | 78.9 | 71.6 | 69.8 |
| TEXAS | 115.6 | 102.8 | 107.0 | 95.5 | 108.8 | 98.0 | 94.6 |
| UTAH | 63.8 | 59.0 | 69.1 | 69.1 | 66.3 | 72.6 | 64.7 |
| VERMONT | 34.2 | 31.5 | 14.9 | 12.2 | 17.7 | 9.3 | 14.6 |
| VIRGINIA | 119.4 | 125.9 | 132.8 | 124.6 | 81.2 | 113.0 | 112.8 |
| WASHINGTON | 64.7 | 71.2 | 74.6 | 68.7 | 76.8 | 74.8 | 77.1 |
| WEST VIRGINIA | 79.4 | 84.2 | 78.4 | 65.4 | 57.6 | 59.4 | 53.0 |
| WISCONSIN | 61.4 | 68.5 | 75.3 | 73.6 | 80.9 | 74.8 | 69.9 |
| WYOMING | 19.8 | 22.5 | 12.0 | 25.3 | 23.4 | 47.1 | 50.9 |
