# Supplementary material for: Diffusion of an innovation: growth in video capsule endoscopy in the U.S. Medicare population from 2003 to 2019
Source: BMC Health Serv Res. 2022 Mar 31;22:425. doi: 10.1186/s12913-022-07780-2 (PMC8969398; doi:10.1186/s12913-022-07780-2)
Supplement: Supplementary file 2 — Additional file 2: Table S2. Performance of esophageal capsule endoscopy (CPT 91111) within the Medicare population 2007–2019. [file 12913_2022_7780_MOESM2_ESM.docx]

**Supplemental Table 2.** Performance of esophageal capsule endoscopy (CPT 91111) within the Medicare population 2007-2019.

|  | CPT 91111 (Esophageal Capsule Endoscopy) |
| --- | --- |
| 2007 | 453 |
| 2008 | 363 |
| 2009 | 419 |
| 2010 | 335 |
| 2011 | 272 |
| 2012 | 228 |
| 2013 | 243 |
| 2014 | 202 |
| 2015 | 191 |
| 2016 | 127 |
| 2017 | 113 |
| 2018 | 114 |
| 2019 | 155 |
